# Beyond the Horizon: Switzerland’s Quest for EU Research Framework Re-Integration

**DOI:** 10.3389/ijph.2024.1607839

**Published:** 2024-10-07

**Authors:** Hania Janta, Michael Käser

**Affiliations:** ^1^ Swiss Tropical and Public Health Institute, Allschwil, Switzerland; ^2^ University of Basel, Basel, Switzerland

**Keywords:** Horizon Europe, Switzerland, EU association, EC relations, research collaboration

## Introduction

At the time of writing this piece, Switzerland is considered a non-associated third country in the largest global research and innovation funding programme, the European Union’s (EU) 9th Framework Programme: Horizon Europe (2021–2027). The exclusion of Switzerland, since May 2021, implies that Swiss-based researchers and innovators cannot receive direct EU funding and coordinate EU research consortia. Amid these challenges, this commentary provides a perspective from the Swiss Tropical and Public Health Institute (Swiss TPH), a world-leading institute in global health research, and a key partner of the Swiss School of Public Health (SSPH+). Swiss TPH has a record of accomplishment with 90 EU projects completed over the past 20 years, including 26 that are still ongoing, and a longstanding history of international collaborations spanning more than 80 years. Despite Swiss government transitional measures being in place, the EU’s downgrading of Switzerland has not only significantly impacted the institute’s visibility, knowledge exchange, collaborative power and hence competitiveness but has also caused misunderstanding and uncertainties amongst the research community, administrative burden and financial strains.

## Decade of Uncertainty: Phases of Exclusion

In 2014, a narrowly passed referendum against mass immigration resulted in Switzerland’s exclusion from EU funding (2014–2016). Such status meant loosing access to the European Research Council (ERC) schemes and the right to lead consortia in collaborative projects, considerably limiting participation in the EU Horizon 2020 programme. Specific networking instruments, such as Marie Skłodowska-Curie Actions (MCSA) became out of reach to the Swiss academics. Reduced Switzerland’s presence in Brussels meant lack of capabilities to shape research agendas and influence EU research priorities. At that time, we observed drop-outs from collaborative consortia originally brought together and driven by our institution [1], and at the University of Bern a decrease in funding scale and reputation were noted [2].

Switzerland then gained the status of “partial association” in 2017, allowing Swiss-based researchers to participate in most funding schemes [1]. However, political developments eventually led to the full marginalisation in EU funding, and in 2021, Switzerland received a “third country status.” The damaging consequences of the exclusion from the Horizon Europe programme, compared to the Horizon 2020 programme, were immediately noted by Swiss-based researchers. Unable to lead the consortia, Swiss researchers have been relying on the goodwill of their collaborators, rather their expertise [3], while bureaucratic hurdles have muddled the grant application process [4]. One considerable consequence of the exclusion from MCSA was the inability of SSPH+ to reapply for an extension of its outstanding stellar international PhD fellowship program GlobalP3HS [5]. This highly competitive PhD program had allowed 50 excellent overseas PhD students to enroll at one of the Swiss universities behind the SSPH+ Foundation. The unavailability of individual grant options in the mid-term leads to a brain drain of world leading researchers, culminating in economic decline and job losses. Overall, the exclusion from EU research programmes jeopardises Switzerland’s international reputation, posing potential long-term challenges in accessing European funding and maintaining prestige. Lavenex and Veuthey [6], succinctly put it: “the association model, once a ‘side-street’ to European integration, is now a ‘dead-end’ (p. 351).”

## Navigating EU Red Tape and Mitigation Measures

In response to the limited access to the EU funds, the Swiss National Funding Agency (SNSF) launched several schemes to remedy inaccessible calls, including European and global bilateral agreements, which facilitate collaboration with various countries and regions (e.g., WEAVE or LEAD), as well as alternatives to the prestigious ERC (SNSF Starting, Consolidator, and Advanced Grants). Of course, although financially competitive, these substitutes lack international recognition, which disadvantage outstanding Swiss researchers and their long-term careers – a point noted by the Swiss-based SNSF-funded researchers in the national survey [3].

Yet, this “dead-end” association model to the Horizon Europe funding does not entirely prevent Switzerland from participating in collaborative projects. Under certain conditions, Switzerland can take part in around two-thirds of the calls for proposals. The EU terminology, however, does not ease the overall confusion among Swiss and non-Swiss researchers: while Switzerland is not an *“associated country*,” a Swiss organisation is considered an “*associated partner”* (from a third country) - and that is not always straightforward to convey to an international team forming a research consortium. From an institutional perspective, Swiss TPH has invested in providing steady information and briefings to explain the eligibility criteria that vary by instrument and call, and from deadline to deadline, to potential collaborators. This tedious task requires a mix of negotiation, communication, and lobbying skills, slowing down grant submission preparation, discouraging Swiss-based researchers from joining an EU consortium for the first time and causing lost opportunities due to frequent non-submissions after lengthy application preparations.

The confusing EU jargon extends throughout the submission process to the grant and consortium agreement negotiations to further complicate matters. In a collaborative consortium, Switzerland is not a “*beneficiary*,” and hence lacks of viewing and editing rights on the EU portal. The administrative burden is then passed onto the consortium lead who needs to place the *“Swiss budget”* as an additional funding source under *“financial contributions”* at an undefined place in a project proposal. If successful, the “*Swiss budget*” is then provided by the Swiss government. Despite this administrative challenge and widespread confusion about Switzerland’s status in an EU consortium, our organisation has, in certain situations, suddenly become highly sought after due to the perception that the Swiss financial contribution to the overall European budget can be more competitive, as “the money comes on top.” This can also cast Swiss organisations in a questionable light.

A key funding scheme for Swiss TPH, the European and Developing Countries Clinical Trials Partnership (EDCTP3), supports clinical research on poverty-related infectious diseases in sub-Saharan Africa and enables collaborations with low- and middle-income countries (LMICs). These countries are eligible for EU funding as “*other third countries*,” allowing them to take on the role of scientific lead. Here, we have observed a notable trend of reverse partnership inclusion by LMIC collaborators incorporating our Swiss research institution as a non-member country partner into their sub-Sahara Africa driven consortia. This brings an interesting equal-partnership along as an unintended [by the European Commission (EC)] outcome, with a decolonisation aspect, and depicts one of the rare positive experiences of the inclusion/exclusion criteria for Horizon Europe – humbling and affirming at the same time. The process to reach this point of collaborative spirit, however, involved decades of global living partnership based on mutual trust, mutual learning for change, and a continued on-the-project training.

## Progress in Talks

The overall outrage with the degradation of Switzerland (and the UK), was demonstrated through a number of national and European campaigns. Launched in 2022, the *Stick to Science* campaign [7] urging the EU, the UK and Switzerland to reach association agreements and to contribute to Horizon Europe, gathered 6000+ signatures, including those from Nobel laureates. The theme of the EU’s inclusion versus exclusion plays a role in positioning Switzerland within the European research landscape. In January 2024, the UK re-joined EU research programmes as an *“associated country*,” exacerbating Switzerland’s isolation and complicating its efforts to re-enter EU funding. At the time of writing, 19 countries are associated with EU research programmes, including some geographically distant from Europe, such as South Korea (from 2025 on) or Tunisia [8]. Given Switzerland’s exclusion, the EU’s negotiations with third countries have raised concerns among European researchers and the lack of transparent communication and inadequate EC web resources have been widely questioned [9].

To what extent these campaigns led to a political change is unclear, nevertheless, on 18 March 2024 the EC and Switzerland reached a Common Understanding, offering hope for Switzerland’s future association to Horizon Europe [10]. A first positive sign of negotiation progress was the decision to allow Swiss researchers to apply for prestigious ERC grants from August 2024 onwards, even before full association with Horizon Europe is agreed upon, offering relief to the Swiss academic community. We sincerely seek for further inclusion of Switzerland into the EDCTP, the MSCA and the entire Horizon Europe including its subsequent programme. As we continue to observe political developments (which lead to even more confusion), Swiss TPH remains both hopeful and cautiously optimistic about re-joining the global academic community as a visible partner. We look forward to making up for the suffered losses, fully leveraging our knowledge, innovation expertise, research skills, and scientific leadership competencies, and resuming our full contribution to cutting-edge global health research.
